# The ability of a new continuous cardiac output monitor to measure trends in cardiac output following implementation of a patient information calibration and an automated exclusion algorithm

**DOI:** 10.1007/s10877-012-9384-7

**Published:** 2012-08-02

**Authors:** Hironori Ishihara, Yoshihiro Sugo, Masato Tsutsui, Takashige Yamada, Tetsufumi Sato, Toshimasa Akazawa, Nobukazu Sato, Koichi Yamashita, Junzo Takeda

**Affiliations:** 1 Department of Anesthesiology, Hirosaki University Graduate School of Medicine, 5 Zaifu-Cho, Hirosaki-Shi, 036-8562 Japan; 2 Monitoring Technology Center, Nihon Kohden Corporation, Tokyo, Japan; 3 Department of Anesthesiology, National Defense Medical College, Saitama, Japan; 4 Department of Anesthesiology, School of Medicine, Keio University, Tokyo, Japan; 5 Department of Anesthesiology and Resuscitology, Okayama University Medical School, Okayama, Japan; 6 Department of Anesthesiology and Pain Medicine, Juntendo University School of Medicine, Tokyo, Japan; 7 Department of Anesthesiology, Toho University, Tokyo, Japan; 8 Department of Anesthesiology and Critical Care Medicine, Kochi Medical School, Kochi, Japan

**Keywords:** Cardiac output, Measurement technique, Non-invasive calibration, Pulse contour analysis, Pulse wave transit time

## Abstract

A new non-invasive continuous cardiac output (esCCO) monitoring system solely utilizing a routine cardiovascular monitor was developed, even though a reference cardiac output (CO) is consistently required. Subsequently, a non-invasive patient information CO calibration together with a new automated exclusion algorithm was implemented in the esCCO system. We evaluated the accuracy and trending ability of the new esCCO system. Either operative or postoperative data of a multicenter study in Japan for evaluation of the accuracy of the original version of esCCO system were used to develop the new esCCO system. A total of 207 patients, mostly cardiac surgical patients, were enrolled in the study. Data were manually reviewed to formulate a new automated exclusion algorithm with enhanced accuracy. Then, a new esCCO system based on a patient information calibration together with the automated exclusion algorithm was developed. CO measured with a new esCCO system was compared with the corresponding intermittent bolus thermodilution CO (ICO) utilizing statistical methods including polar plots analysis. A total of 465 sets of CO data obtained using the new esCCO system were evaluated. The difference in the CO value between the new esCCO and ICO was 0.34 ± 1.50 (SD) L/min (95 % confidence limits of −2.60 to 3.28 L/min). The percentage error was 69.6 %. Polar plots analysis showed that the mean polar angle was −1.6° and radial limits of agreement were ±53.3°. This study demonstrates that the patient information calibration is clinically useful as ICO, but trending ability of the new esCCO system is not clinically acceptable as judged by percentage error and polar plots analysis, even though it’s trending ability is comparable with currently available arterial waveform analysis methods.

## Introduction

Continuous cardiac output (CCO) measurement calculated by a thermodilution method (CCOpa) utilizing pulmonary artery catheter measurements is being used to optimize cardiovascular management of critically ill patients. However, it would be clinically useful to measure CCO solely using routine cardiovascular monitors, without any need for further sensors or procedures. A novel non-invasive continuous cardiac output (esCCO) measurement method solely utilizing the routine clinical monitor, based on pulse contour analysis combined with pulse wave transit time (PWTT) was devised by Sugo et al. [1], and this method was demonstrated to have the potential to give rise to an alternative non-invasive cardiac output (CO) trend for post-cardiac surgical patients without apparent arrhythmia [2]. PWTT is the sum of pre-ejection period (PEP) and pulse wave arrival time from the ascending aorta to the peripheral pulse oximetry (SpO_2_) probe site, and its changes have been reported to predict changes in blood pressure [3]. PWTT is calculated from the interval between R wave of ECG and peripheral SpO_2_ pulse wave arrival when ECG and SpO_2_ are simultaneously recorded. A multicenter study involving seven university hospitals in Japan was performed to test the accuracy of this method, mostly during and after cardiac surgery, including off-pump coronary artery bypass grafting [4]. The results of the multicenter study were comparable with those of the previous study [2], supporting the clinical relevance of this method not only in the ICU, but also during surgical procedures. Thereafter, an automated exclusion algorithm for the esCCO system was further developed to promote the reliability of the measurement, even though its limitations became evident during some surgical procedures, such as cardiopulmonary bypass (CPB). Furthermore, as the esCCO system consistently requires a reference CO at the start of measurement, a non-invasive CO derived from patient demographic data and conventional cardiovascular variables was developed. We examined whether the new esCCO system based on a patient information calibration together with a new automated exclusion algorithm would have acceptable trending ability.

## Methods

Data of a multicenter study involving seven university hospitals in Japan for evaluation of the accuracy of the original version of esCCO system [4] were used to develop the new esCCO system. The study was conducted with the approval of the institutional review board of each of the participating institutions, and with written informed consent from each patient obtained before the surgery. Patients with significant tricuspid valve insufficiency were excluded from the study, as a high degree of tricuspid regurgitation can cause underestimation of the thermodilution CO [5]. A total of 213 patients were initially enrolled in the study. Six patients with incomplete datasets were excluded from the study. Most were cardiac surgical patients who had complete sets of cardiovascular data that did not indicate continued arrhythmia. Among these, 133 patients were also evaluated postoperatively in the intensive care unit (ICU), and 74 patients were evaluated while undergoing anesthesia and surgery in the operating room (Table 1). esCCO was computed continuously using an ECG monitor, an arterial pressure monitor, and a pulse oximetry monitor. A balloon tipped flow-directed thermodilution pulmonary artery catheter (Swan-Ganz CCOmbo CCO/SVO_2_, ref. 744HF75, Baxter Healthcare, Edwards Critical Care Division, Irvine, CA, USA) was inserted before the start of surgery after induction of anesthesia. Correct positioning was confirmed by pressure tracing as well as chest radiography. The catheter was connected to a CCOpa monitor (Vigilance, Version 5.4, Baxter Healthcare, Irvine, CA, USA). Subsequently, both CCOpa and esCCO measurements were started. As esCCO consistently requires a reference CO value obtained utilizing another CO measurement system, a corresponding intermittent bolus thermodilution CO (ICO) value was used as a reference when relatively stable cardiovascular states were achieved soon after admission to the ICU or after insertion of the catheter before the start of surgery in the operating room. Thereafter, esCCO was periodically compared with ICO either at 10 a.m. on subsequent postoperative days until discontinuation of the thermodilution method for CO measurement in the ICU or at one-hour intervals after the first measurement before the start of surgery until the end of surgery in the operating room except during cardiopulmonary bypass. The ICO was measured following a bolus injection of 10 ml chilled isotonic saline (<8 °C) through the injectate port of the pulmonary artery catheter. Measurements were performed in triplicate at random points in the respiratory cycle. A variation of ±10 % within triplicate measurements was defined as acceptable.

**Table 1:** Patient characteristics

Site	ICU	OR	Total
No. of patients	133	74	207
Sex (m/f)	90/43	48/26	138/69
Age (years)	65.9 ± 11.2	64.0 ± 14.8	65.3 ± 12.6
Height (cm)	159.4 ± 9.9	160.9 ± 10.9	159.9 ± 10.3
Body weight (kg)	59.3 ± 13.0	58.7 ± 11.4	59.1 ± 12.5
BSA (m^2^)	1.60 ± 0.20	1.61 ± 0.19	1.61 ± 0.20
Duration (h)	36.4 ± 26.0	7.6 ± 2.9	26.1 ± 25.0
Range of CO (l/min)	1.5–15.5	1.3–13.1	1.3–15.5
Operative procedure (No. of patients)	Cardiac surgery (124) Liver transplantation (9)	Cardiac surgery (50) Kidney transplantation (11) Hepatic segmentectomy (3) Abdominal aortic surgery (3) Pancreatectomy (7)	

Age, Height, Body weight, BSA, and Duration are presented as mean ± standard deviation. The CO range is presented as minimum–maximum

*ICU* intensive care unit, *OR* operating room, *BSA* body surface area, *Duration* duration of measurement

### New automated exclusion algorithm

**Fig. 1:**
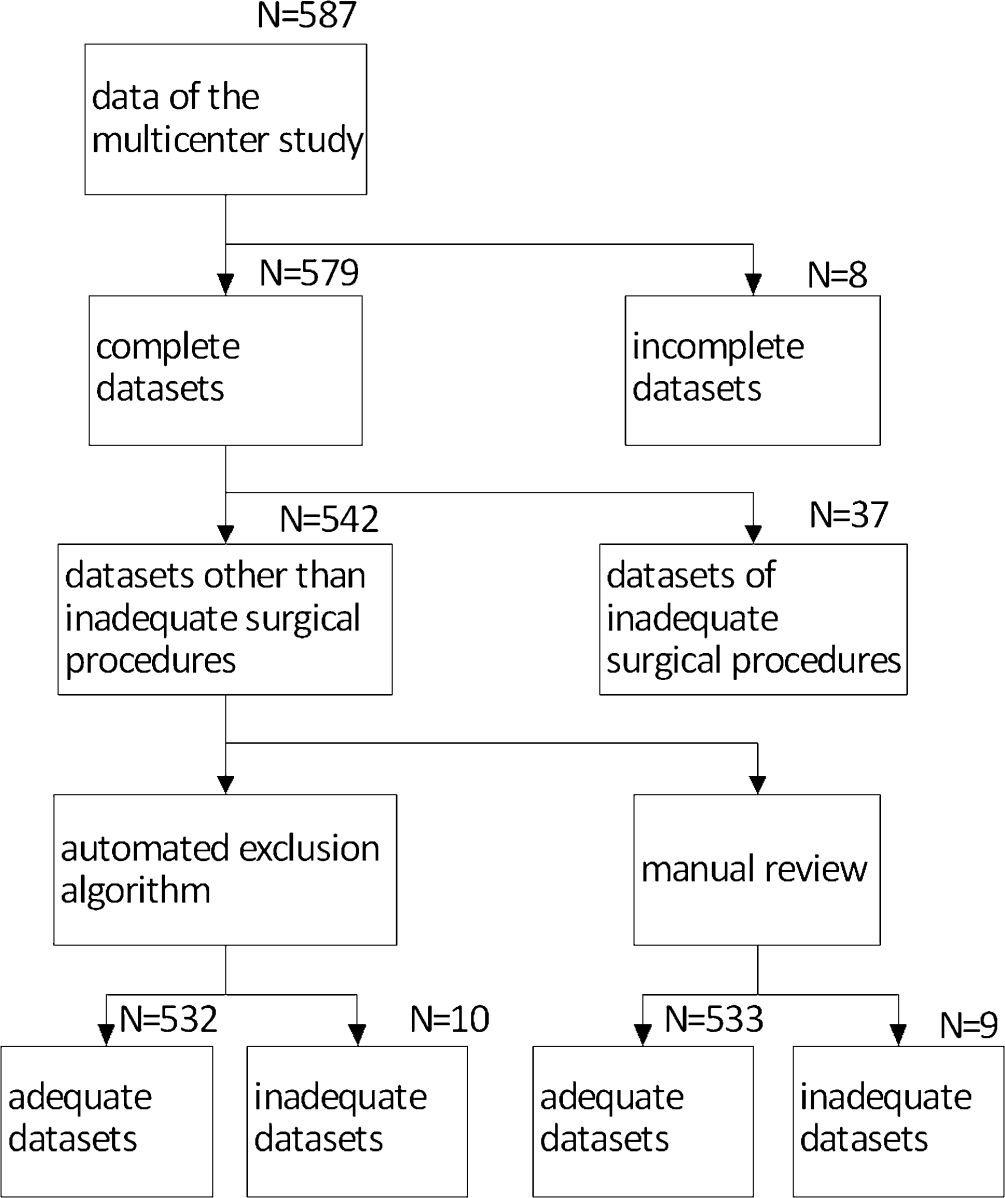
Data flow diagram of manual review and automated exclusion

Prior to applying a patient information calibration, a new automated exclusion algorithm was formulated to promote the accuracy of the original version of the esCCO system. A total of 587 paired sets of data of the multicenter study were reviewed by one author (YS). Thereafter, a new exclusion algorithm was developed from 542 datasets for the heart rate, pulse pressure, esCCO, including PWTT, and ICO, after 37 theoretically inadequate datasets collected during the some surgical procedures such as CPB, had been excluded as shown in Fig. 1.

### Patient information calibration for esCCO

After confirming the adequacy of each measurement data, the new esCCO system was developed using a patient information CO calibration together with the new automated exclusion algorithm. Multiple regression analysis was conducted to formulate the patient information CO calibration using patient demographic data, including age, gender, height, and body weight as well as blood pressure, heart rate and PWTT at the corresponding time-points (“Appendix 1”). The result was applied as the reference for the new esCCO system, instead of ICO. Fifty-four patients (28 patients from one institution (NDMC) seen from September 2007 to January 2008, and 26 patients selected through multi-facility evaluation from November 2007 to February 2008) served as the training set for use as the reference for the new esCCO system. Both maximum and minimum ICO data for each patient were used for the multiple regression analysis.

Among the total of 213 patients undergoing multicenter evaluation, 26 patients were recruited for the training set, and six patients with incomplete sets of independent variables were excluded from the study. Consequently, the remaining 181 patients served as the validation set.

### Statistical analysis

Data are expressed as mean ± SD. Bland-Altman analysis was used to compare the esCCO and ICO. Statistical analysis using Bland-Altman analysis associated with multiple measurements correction was also performed for esCCO data applying the new exclusion algorithm compared with ICO [6]. Regression analysis of both was also performed. The percentage error was calculated as the ratio of 2SD of the bias to the mean ICO and was considered clinically acceptable if it was 30 % or less, as proposed by Critchley and Critchley [7]. Furthermore, the trending ability of changes in the esCCO system was assessed by using the polar plots method proposed by Critchley et al. [8, 9]. Changes in CO values were calculated by subtracting the previous CO value from the current CO value. When ICO is the reference method, they would recommend an angular bias no greater than ±5° and radial limits agreement no greater than ±30° for good trending ability. Statistically significant difference was considered at *p* < 0.05.

## Results

### Manual review and automated exclusion

Thirty-seven data during aortic cross clamping and during construction of posterior wall grafts in off-pump coronary artery bypass (OPCAB) as well as CPB were found to be theoretically inadequate for esCCO measurement, due to changes in the SpO_2_ and/or ECG waveforms. Thereafter, either manual review or the new automated exclusion algorithm was applied in the remaining 542 datasets. Eight datasets were manually judged to be inadequate due to erroneous R wave detection. One dataset obtained during postoperative cardiac tamponade was also manually excluded due to the abnormal relationship between the SV and PWTT (the SV was 39.7 ml, and the PWTT, 199 ms before cardiac tamponade, and the corresponding values were 13.6 ml and 157 ms during the cardiac tamponade). The difference between the original esCCO data following manual review and the ICO was 0.08 ± 1.07 L/min, with a linear correlation between the two CO measures (r = 0.82, n = 533, *p* < 0.01). The percentage error was 47.2 %.

**Fig. 2:**
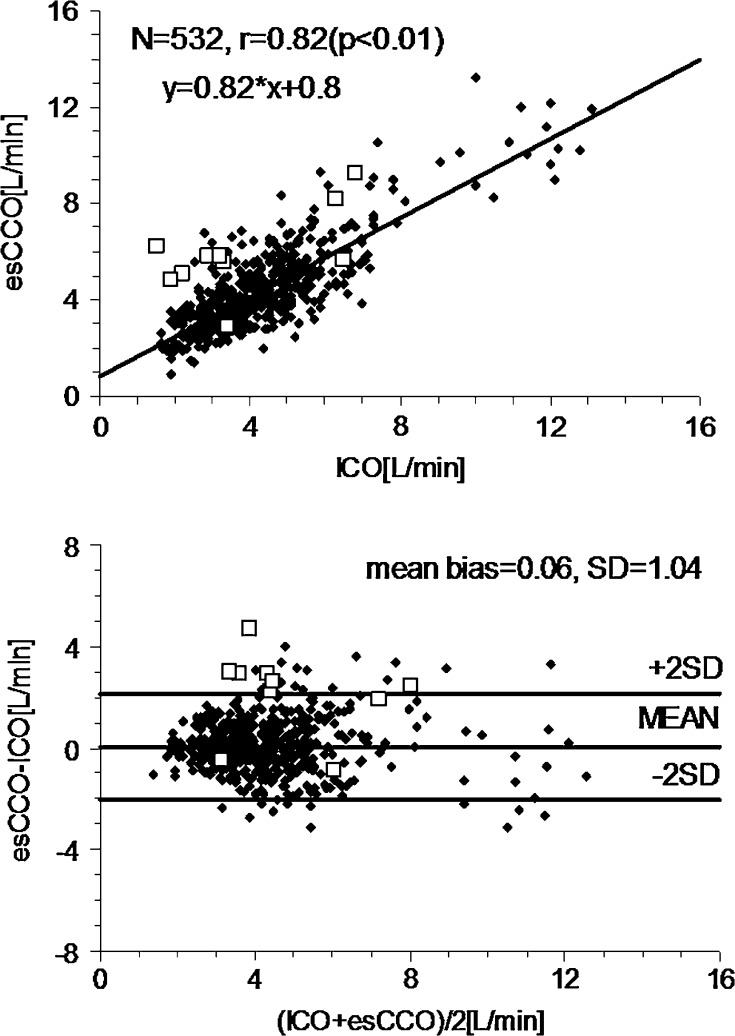
Scatter plots between intermittent bolus cardiac output (ICO) and estimated continuous cardiac output (esCCO) after implementation of the new automated exclusion algorithm (*upper*), along with Bland-Altman plots (*lower*). *Closed diamonds* indicate adequate data (n = 532). *Open squares* indicate inadequate data as judged by the new exclusion algorithm (n = 10)

The new automated exclusion algorithm judged ten datasets including one dataset obtained during cardiac tamponade as inadequate. The difference between the original esCCO together with the new exclusion algorithm and the ICO was 0.06 ± 1.04 L/min, with a linear correlation between the two (r = 0.82, n = 532, *p* < 0.01) (Fig. 2). The SD associated with multiple measurements correction was 1.05L/min. The percentage error was 48.5 %. In the original esCCO system an angular bias using polar plots was −1.8° and radial limits of agreement were ±53.3°. Polar concordance rate at 30° was 76.2 %.

### The new esCCO system based on the patient information calibration

**Fig. 3:**
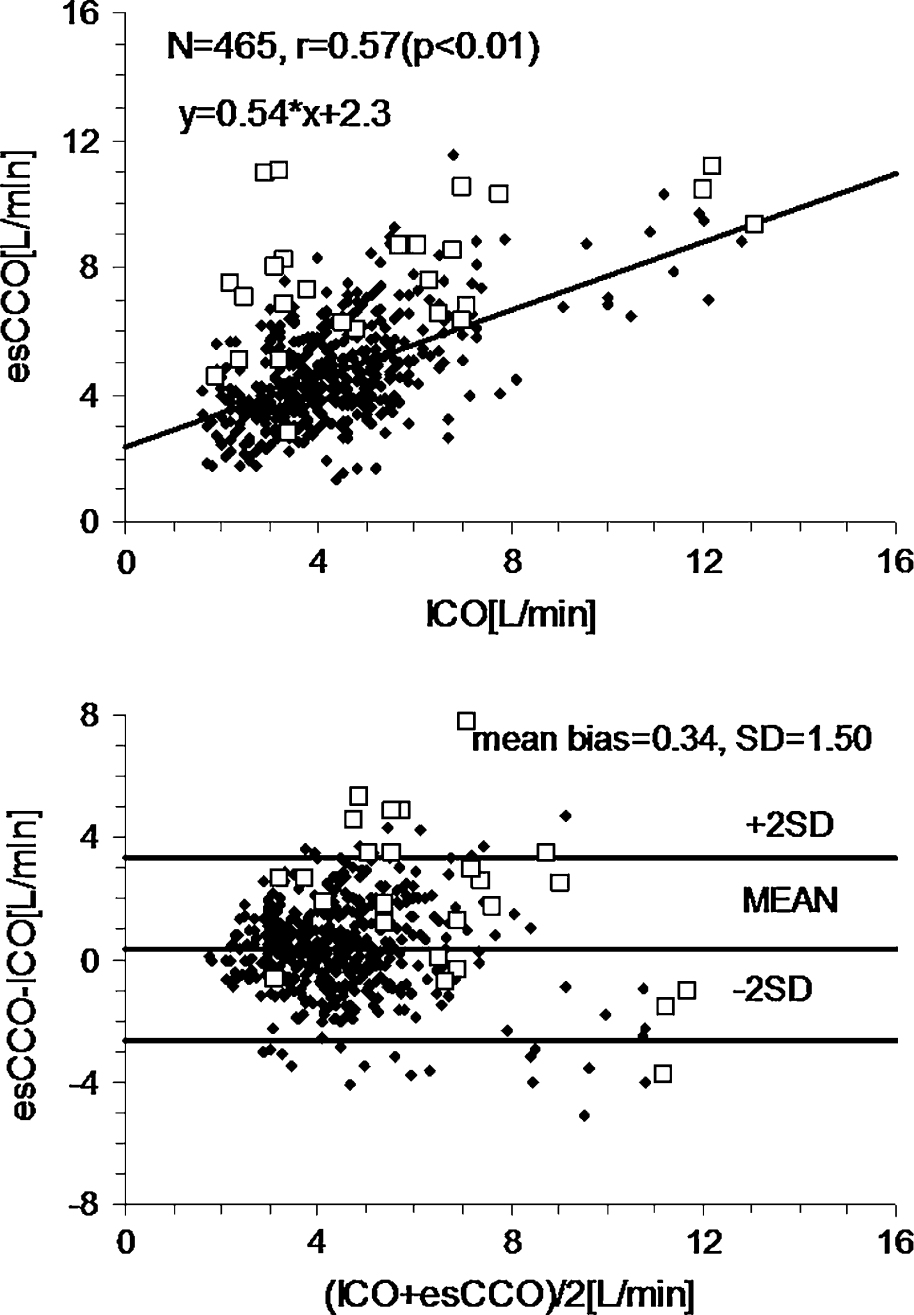
Scatter plots between intermittent bolus cardiac output (ICO) and estimated continuous cardiac output (esCCO) using a patient information calibration together with a new automated exclusion algorithm (*upper*); along with Bland-Altman plots (*lower*). *Closed diamonds* indicate adequate data (n = 465). *Open squares* indicate inadequate data judged by the new exclusion algorithm (n = 26)

**Fig. 4:**
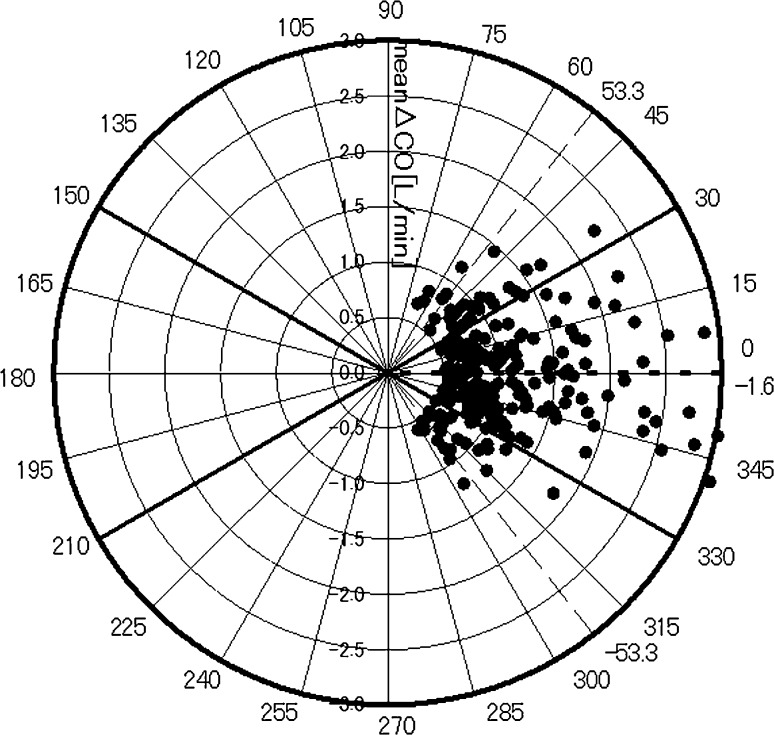
Polar plots showing the trending ability of the new esCCO system. *Half*-*circle* polar plots are shown with data transformed to positive directional data only (n = 230) without central zone data (<0.5 L/min, n = 205). The distance from the center of the plot represents the mean change in cardiac output. The angular bias is −1.6°. The dashed lines of ±53.3° represent the limits of agreement. Polar concordance rate at 30° is 75.2 %

The difference between the new esCCO and ICO in the validation study was 0.15 ± 1.47 L/min at the calibration point, and 0.34 ± 1.50 L/min at the subsequent measurement points. The SD associated with multiple measurements correction was also 1.50 L/min. There was a linear correlation between the two (r = 0.64, n = 181, *p* < 0.01 for the former and r = 0.57, n = 465, *p* < 0.01 for the latter, respectively) (Fig. 3), and the percentage error was 69.6 %. In the new esCCO system the mean polar angle was −1.6° and radial limits of agreement were ±53.3°. Polar concordance rate at 30° was 75.2 % (Fig. 4).

## Discussion

Although data collected during the three surgical procedures in which limitations of accurate measurement became evident had been excluded before the development of the new automated exclusion algorithm, the results of this study suggest that the new automated exclusion algorithm is as effective as a retrospective manual review, as judged by the Bland-Altman plots and percentage error, even though 26 CO data were excluded by the automated exclusion algorithm in the new esCCO system, since its threshold level was set lower compared to the original esCCO system.

The reported percentage error from several studies for CO in arterial waveform analysis using standard radial artery catheterization (APCO) such as the FloTrac/Vigileo monitor (Edwards Lifescience, Irvin, USA) is in the range of approximately 35–60 % [10–12]. Considering a percentage error of 48.5 % for the original esCCO system with the new automated exclusion algorithm, the original esCCO system with ICO calibration cannot be interchangeable with ICO, but would be comparable with the FloTrac/Vigileo monitor.

Critchley et al. [8], however, have recently pointed out problems of the analytical methods, including use of the Bland-Altman plots and percentage error, since the magnitude of the underlying CO change and the degree of agreement are totally ignored. To overcome these problems for assessment of its trending ability, the same authors have proposed a new approach using polar plots analysis [8], and have further developed the methodology and devised initial guidelines for decision making on the performance of new CO monitors [9]. When ICO is used as the reference method, they would recommend an angular bias of no greater than ±5° and radial limits of agreement of no greater than ±30° for good trending ability. Either the original or the new esCCO system had an angular bias was less than 5°, but radial limits were apparently greater than ±30°, indicating that the calibration method of the esCCO system is good, but the trending ability is poor.

The reported an angular bias and radial limits of agreement for APCO using the FloTrac/Vigileo monitor are the range of −33 to 8° for the former and of ±40 to 50° for the latter, respectively [9], suggesting that trending ability of the new esCCO system is comparable with the FloTrac/Vigileo monitor.

There would be several potentially undesirable measurement conditions present for acceptable trending ability in this study. Firstly, changes in the SpO_2_ probe site and body position may occur during routine patient care particularly in the ICU. These changes which were not consistently recognized in data review process may have a significant impact on PWTT values, and therefore trending ability of esCCO system.

Secondly, the esCCO system may be relatively accurate even in the presence of changes in the systemic vascular resistance (SVR) [2, 3, 13]. However, the result of a recent multicenter study showed that SVR slightly affected the accuracy of the system [4]. Therefore, apparent changes of SVR after the first CO calibration may have a significant impact on esCCO values in some patients of this study.

Thirdly, it appears that inaccuracy of the esCCO system can occur not only during CPB or aortic cross clamping, but also after aortic cross declamping, after discontinuation of cardiac pacing, or shortly after weaning from CPB, as observed for APCO [14]. Kanazawa et al. [15] reported a decrease in the aortic elasticity after CPB, associated with an aortic-to-radial arterial pressure gradient. Although precise pathophysiology of these inaccuracies remains unclear, a decrease in the aortic elasticity would affect either the PWTT or the peripheral pulse pressure, leading to transient inaccuracy of the esCCO soon after the weaning from CPB. Presumably, there would be additional inadequate esCCO data, even after some of them had been excluded by the automated exclusion algorithm.

Lastly, sampling size of this study for polar plots analysis was the largest (230 inclusion points) when compared with other studies of CO measurement methods described in a report by Critchley et al [9]. Patients in this study were recruited mostly from cardiac surgery, but also other various major surgery as described in Table 1. Therefore, the heterogeneity of the patient population may affect the result of this study at least partly.

Multiple regression analysis was performed to calculate the patient information calibration as the reference for the esCCO. The patient demographic data, heart rate, pulse pressure, and PWTT were used for this purpose. Heart rate, mean arterial pressure, and standard deviation of the arterial pressure have a major impact on the determination of APCO [16]. Additionally, the patient’s specific vascular impedance and compliance calculated from the patient demographic data, including age, sex, height and body weight also plays a significant role in determining the CO [10]. Furthermore, PWTT is added to calculate the patient information calibration, since pulse wave velocity measured by the arterial pressure waveform at carotid and radial arteries was inversely correlated with the stroke volume divided by the pulse pressure [17]. The result of polar plots analysis would support the usefulness of the patient information calibration in the new esCCO system.

There are several limitations to this study, in addition to undesirable measurement conditions described above as well as theoretically inadequate data collection during the ECG or pulse oximetry probe displacement, arrhythmias, intra-aortic balloon pumping, and/or certain surgical procedures. When a wide QRS complex or a high T wave is present, the present version of the esCCO system fails consistent and accurate R wave detection. Therefore, further amendment of the software is mandatory. In addition, this study did not consistently take other unknown undesirable conditions that may potentially exert influence into consideration during the new esCCO measurement, since this study was aimed at evaluating the new esCCO system during routine patient care rather than in research-oriented management. Clinical esCCO data during body position change, increased intrathoracic pressure, volume loading and apparent vasoconstrictive states are lacking, even though such factors could easily influence peripheral arterial pressure registration [10]. Therefore, further prospective studies of the esCCO system are required to test the effectiveness of the esCCO system in cases involving different pathophysiologies.

In conclusion, this study demonstrates that limitations of the esCCO system become evident during some cardiac surgical procedures, that the patient information calibration is clinically useful as ICO, but trending ability of the new esCCO system is not clinically acceptable based on percentage error and polar plots analysis, even though its trending ability is comparable with currently available arterial waveform analysis methods.

## Appendix 1

Multiple regression analysis was conducted using SPSS II for Windows ver.11.0.1 J (SPSS Inc. Chicago, IL, USA). ICO was defined as a dependent variable, whereas heart rate (HR), pulse pressure (PP), PWTT, age, gender, height, weight, and body surface area (BSA) were defined as independent variables. One to a maximum of 10 pairs of each dependent variable were used to find out the best-fit formula, and 7 types of formulae were prepared. For each formula, the SSe (residual sum of squares), AIC (Akaike’s information criterion), residual minimum and maximum values, and standard deviation were checked, and finally the following Formula (1) was obtained as a regression formula that shows a stable model.

1 $$ {\text{CO}} = {\text{a}} + {\text{b}}*{\text{Gender}} + {\text{c}}*{\text{Age}} + {\text{d}}*{\text{BSA}} + {\text{e}}*{\text{PWTT}} + {\text{f}}*{\text{HR}}*{\text{PP}} $$

CO = cardiac output (L/min); Gender: male = 0, female = 1; Age: years; BSA = body surface area (m^2^); PWTT = pulse wave transit time (msec); HR = heart rate (rpm); PP = pulse pressure (mmHg). These cardiovascular variables are the values, respectively, at the calibration point. a, b, c, d, e, and f are constants or coefficients determined by conducting multiple regression analysis using learning data.
